# Cobalt-doped bioceramic scaffolds fabricated by 3D printing show enhanced osteogenic and angiogenic properties for bone repair

**DOI:** 10.1186/s12938-021-00907-2

**Published:** 2021-07-24

**Authors:** Jungang Li, Chaoqian Zhao, Chun Liu, Zhenyu Wang, Zeming Ling, Bin Lin, Bizhi Tan, Linquan Zhou, Yan Chen, Delong Liu, Xuenong Zou, Wenge Liu

**Affiliations:** 1 Department of Orthopaedics, Fujian Medical University Union Hospital, Fuzhou, 350001 China; 2 Key Laboratory of Optoelectronic Materials Chemical and Physics, Fujian Institute of Research on the Structure of Matter, Chinese Academy of Sciences, Fuzhou, China; 3 Guangdong Provincial Key Laboratory of Orthopaedics and Traumatology, Department of Spine Surgery, The First Affiliated Hospital of Sun Yat-Sen University, Guangzhou, 510080 China

**Keywords:** Cobalt, Bioceramics, Angiogenesis, Osteogenesis, Bone defect repair

## Abstract

**Background:**

The bone regeneration of artificial bone grafts is still in need of a breakthrough to improve the processes of bone defect repair. Artificial bone grafts should be modified to enable angiogenesis and thus improve osteogenesis. We have previously revealed that crystalline Ca_10_Li(PO_4_)_7_ (CLP) possesses higher compressive strength and better biocompatibility than that of pure beta-tricalcium phosphate (β-TCP). In this work, we explored the possibility of cobalt (Co), known for mimicking hypoxia, doped into CLP to promote osteogenesis and angiogenesis.

**Methods:**

We designed and manufactured porous scaffolds by doping CLP with various concentrations of Co (0, 0.1, 0.25, 0.5, and 1 mol%) and using 3D printing techniques. The crystal phase, surface morphology, compressive strength, in vitro degradation, and mineralization properties of Co-doped and -undoped CLP scaffolds were investigated. Next, we investigated the biocompatibility and effects of Co-doped and -undoped samples on osteogenic and angiogenic properties in vitro and on bone regeneration in rat cranium defects.

**Results:**

With increasing Co-doping level, the compressive strength of Co-doped CLP scaffolds decreased in comparison with that of undoped CLP scaffolds, especially when the Co-doping concentration increased to 1 mol%. Co-doped CLP scaffolds possessed excellent degradation properties compared with those of undoped CLP scaffolds. The (0.1, 0.25, 0.5 mol%) Co-doped CLP scaffolds had mineralization properties similar to those of undoped CLP scaffolds, whereas the 1 mol% Co-doped CLP scaffolds shown no mineralization changes. Furthermore, compared with undoped scaffolds, Co-doped CLP scaffolds possessed excellent biocompatibility and prominent osteogenic and angiogenic properties in vitro, notably when the doping concentration was 0.25 mol%. After 8 weeks of implantation, 0.25 mol% Co-doped scaffolds had markedly enhanced bone regeneration at the defect site compared with that of the undoped scaffold.

**Conclusion:**

In summary, CLP doped with 0.25 mol% Co^2+^ ions is a prospective method to enhance osteogenic and angiogenic properties, thus promoting bone regeneration in bone defect repair.

**Supplementary Information:**

The online version contains supplementary material available at 10.1186/s12938-021-00907-2.

## Background

Currently, the reconstruction of bone defects associated with tumors, trauma, inflammation, and surgery represents a challenge for clinicians in orthopedic practice. To date, autologous bone grafts are still considered the optimal standard for the regeneration of bone defects [1]. However, disadvantages, such as inadequate bone sources and donor site morbidity, have limited its applications in practice. Allogeneic and xenogeneic bone grafts, as an alternative to autografts, can avoid these shortcomings mentioned above. However, the high risks of immunological rejection and pathogen transmission impose a major restriction on their clinical application [2, 3].

Artificial bone grafts have been developed to solve the above problems due to their advantages of readily availability, superior stability, and desired reproducibility [4]. Standing out among artificial bone grafts, bioceramics, particularly beta-tricalcium phosphate (β-TCP), exhibit desired biocompatibility, good degradability, and remarkable osteoconductivity and osteoinductivity [5]. β-TCP has recently drawn substantial attention and further become a hopeful artificial bone graft alternative [5, 6]. In vitro studies showed that bone marrow mesenchymal stem cells (BMSCs) seeded on β-TCP were stimulated to proliferate and differentiate into osteoblasts [7, 8]. In vivo trials, with bone remodeling, β-TCP was gradually degraded and eventually displaced by mature new bone [5, 9]. Nevertheless, disadvantages such as poor angiogenic properties severely limit its applications.

Angiogenesis, generally regarded as a challenge for artificial bone grafts, is vital for osteogenesis, and artificial bone grafts should be modified to enable angiogenesis and thus improve osteogenesis [10]. Blood vessels not only recruit osteoprogenitors to the graft surface, but also furnish them with both nutrients and minerals for cell survival and mineralization [11, 12]. Understanding the relationship between angiogenesis and osteogenesis would be of great significance to improve bone regeneration.

Cobalt (Co), known for simulating hypoxic environment, was found to stabilize hypoxia-inducible factor 1-α (HIF-1a) from degradation by inactivating proline hydroxylase (PHD) under normal oxygen pressure. The hypoxic environment can activate HIF-1a pathway cascades and then upregulate the expression of targeted genes such as vascular endothelial growth factor (VEGF) [13]. VEGF is not only beneficial to angiogenesis, but also essential for bone development and regeneration [14]. Modification by doping Co into biomaterials, such as calcium phosphate coatings, hydroxyapatite, and bioactive glass, had positive effects on angiogenesis and osteogenesis in vitro and enhanced bone regeneration in vivo [15–18].

We previously fabricated crystalline Ca_10_Li(PO_4_)_7_ (CLP) by doping lithium (Li) into β-TCP substrate, and the results revealed that CLP possessed relatively higher compressive strength and better biocompatibility than pure β-TCP [19]. To improve the angiogenic properties of CLP, we hypothesized that CLP doped with Co^2+^ ions could achieve excellent osteogenic and angiogenic properties while maintaining a certain compressive strength to promote bone regeneration. In addition to the composition of artificial bone grafts, the structure fabricated by different techniques will also affect its performance. Slurry extrusion 3D printing (SE-3DP) has been applied to manufacture porous scaffolds with precise, dimensionally controlled and orderly spatial microstructures, including porosity properties [20, 21].

**Fig. 1:**
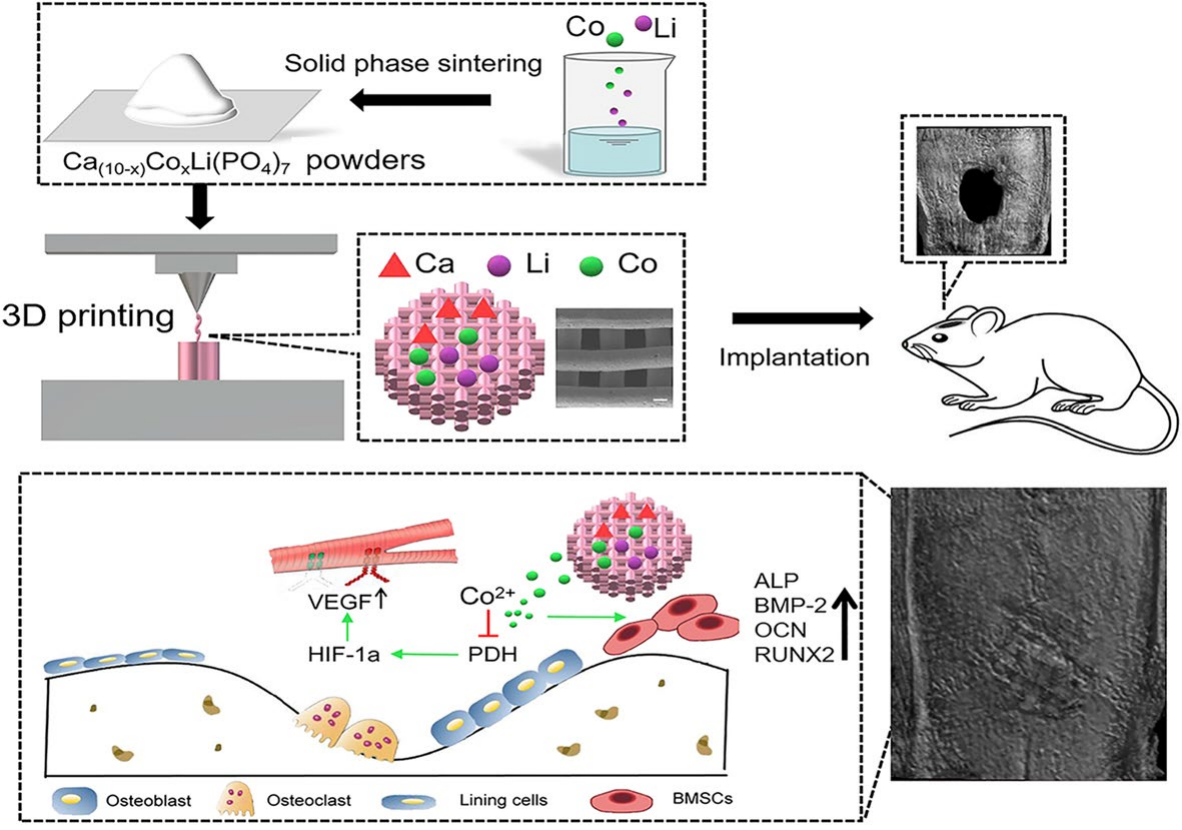
Schematic illustration of the fabrication of porous bioceramic scaffolds doped with different amounts of cobalt to promote bone defect repair via enhanced osteogenic and angiogenic properties

In this study, we investigated the osteogenic and angiogenic effects of CLP doped with Co^2+^ ions. Porous Co-doped CLP scaffolds were prepared by the solid-phase sintering method and SE-3DP techniques [19]. The effects of different Co concentrations in CLP on its physicochemical properties, biocompatibility, and osteogenic and angiogenic properties were systematically investigated in vitro. At 8 weeks after scaffold implantation, microcomputed tomography (micro-CT) and histological methods were utilized to analyze the in vivo bone regeneration of Co-doped scaffolds (as displayed in Fig. 1). We believe that our findings will have great significance for clinical application in the bone repair field.

## Results

### Characterization of the Co (0, 1, 2.5, 5, 10) scaffolds

**Fig. 2:**
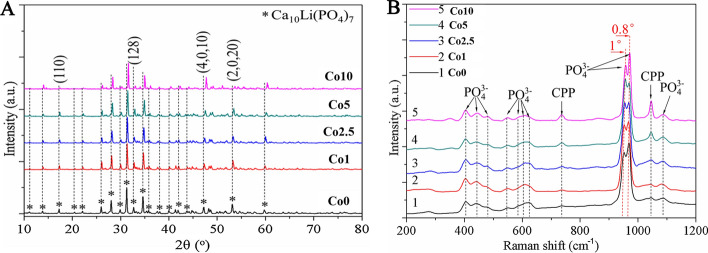
Characterization of the Co (0, 1, 2.5, 5, 10) scaffolds. **A** XRD patterns and **B** Raman spectrum of the Co (0, 1, 2.5, 5, 10) scaffolds

In the present study, Co-doped scaffolds were synthesized by doping CLP with various concentrations of Co (0, 0.1, 0.25, 0.5, 1 mol%) and SE-3D printing techniques, named Co0, Co1, Co2.5, Co5, and Co10 scaffolds. The effect of Co doping on the CLP structure was determined by characterizing the Co (0, 1, 2.5, 5, 10) scaffolds via X-ray diffraction (XRD), Raman spectrophotometry, and scanning electron microscope (SEM). Figure 2A shows the XRD patterns of the Co (0, 1, 2.5, 5, 10) scaffolds. The XRD patterns of the Co (1, 2.5, 5, 10) scaffolds were found to be similar to that of Co0 and were devoid of any additional peaks corresponding to Co. With increasing Co-doping content, the XRD profile moved toward a slight right shift as a whole. When the doping percentage of Co^2+^ ions increased up to 1 mol%, the crystal planes (110), (128), (4,0,10), and (2,0,20) obviously changed.

Figure 2B displays the Raman spectra of the Co (1, 2.5, 5, 10) scaffolds. Compared with the Co0 scaffold, the band positions of the Co (1, 2.5, 5, 10) scaffolds were altered, moving to the direction of a higher Raman shift with increasing Co-doping level.

**Fig. 3:**
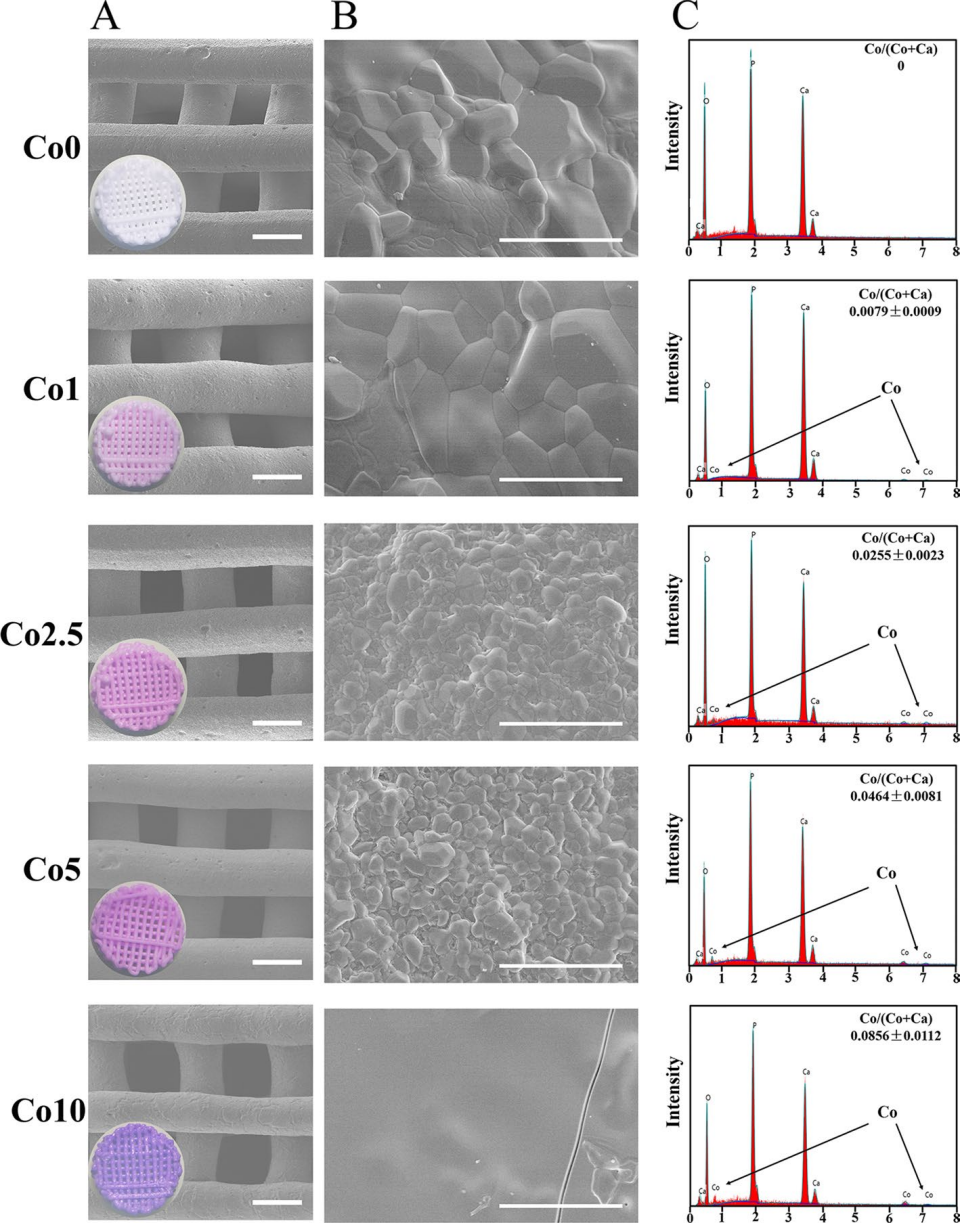
Characterization of the Co (0, 1, 2.5, 5, 10) scaffolds. **A**, **B** SEM images of the surface morphology of the Co (0, 1, 2.5, 5, 10) scaffolds with EDS analysis (**C**) (scale bars: Figure **A** = 500 µm, Figure **B** = 10 µm)

In Fig. 3A, a, color difference was observed between the Co-doped and -undoped scaffolds. After adding Co^2+^ ions to CLP, the color of the scaffolds transformed from white to purple. With increasing Co content, the color of the scaffolds successively changed from white (Co0) to light purple (Co1) to purple (Co2.5) to dark purple (Co5) and to dark reddish purple (Co10). Figure 3A and B demonstrates the SEM surface morphology of the Co (0, 1, 2.5, 5, 10) scaffolds. Based on the micrographs, regular pores and cylindrical wires of scaffolds fabricated by SE-3DP were interlaced in each layer. The printing wire and pore size were approximately 500 μm and 400 μm × 400 μm, respectively. At high magnification (Fig. 3B), the crystal size of the Co (0, 1, 2.5, 5) scaffolds decreased gradually with increasing Co dopant. When the doping percentage was up to 1 mol%, some melted crystals and microcracks were observed on the Co10 scaffold surface. In Fig. 3C, energy-dispersive spectroscopy (EDS) results showed peaks of Co within the Co (1, 2.5, 5, 10) scaffolds. However, there was no characteristic peak of Co in the Co0 scaffold. In addition, the measured Co/ (Ca + Co) ratios enhanced with increasing Co-doping amount. The EDS results also revealed that Co^2+^ ions were successfully incorporated into Co (1, 2.5, 5, 10) scaffolds.

### In vitro* degradation and mineralization properties of the Co (0, 1, 2.5, 5, 10) scaffolds*

**Fig. 4:**
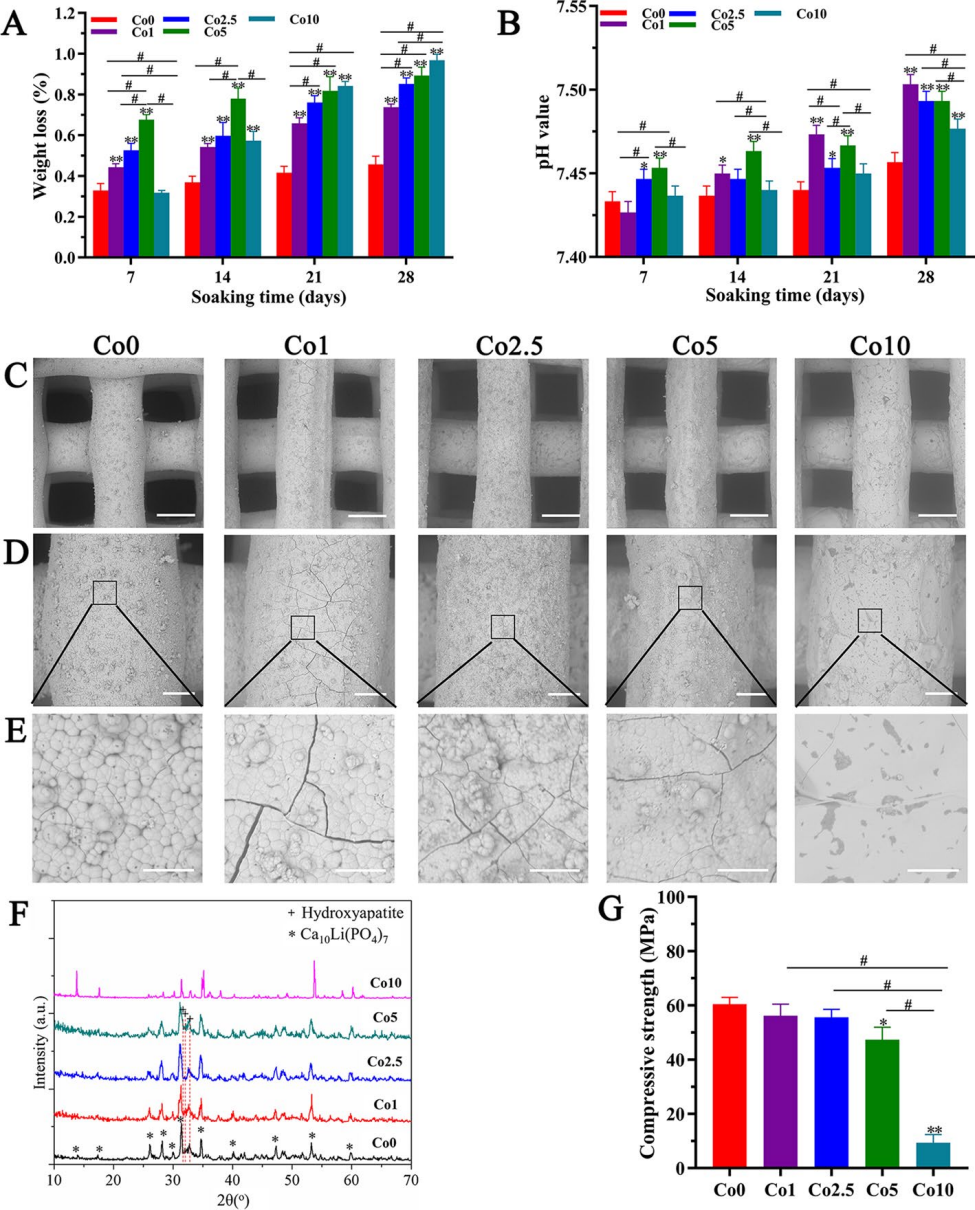
In vitro physicochemical properties of the Co (0, 1, 2.5, 5, 10) scaffolds. **A** Weight loss of the Co (0, 1, 2.5, 5, 10) scaffolds. **B** pH value of Tris–HCl solution after soaking the Co (0, 1, 2.5, 5, 10) scaffolds. **C**–**E** SEM images of the surface microstructure of the Co (0, 1, 2.5, 5, 10) scaffolds after mineralization in vitro (scale bars: Figure **C** = 300 µm, Figure **D** = 100 µm, Figure **E** = 20 µm). **F** XRD patterns of the Co (0, 1, 2.5, 5, 10) scaffolds after mineralization in vitro. **G** Compressive strength of the Co (0, 1, 2.5, 5, 10) scaffolds. The data are expressed as the mean ± SD. *p < 0.05 and **p < 0.01, comparison with the Co0 group; ^**#**^*p* < 0.05, comparison among the Co (1, 2.5, 5, 10) groups

To study the effect of Co doping on the physicochemical properties of CLP, degradation and mineralization tests were performed. Figure 4A and B shows the degradation properties of the Co (0, 1, 2.5, 5, 10) scaffolds in Tris–HCl solution. As shown in Fig. 4A, the weight loss of the Co (0, 1, 2.5, 5, 10) scaffolds tended to increase with the extension of soaking time. At days 21 and 28, the weight loss of the Co (0, 1, 2.5, 5, 10) scaffolds also raised with increasing Co-doping concentration. Meanwhile, the weight loss of the Co (1, 2.5, 5, 10) scaffolds was higher than that of Co0 scaffolds, and the differences were significant (*p* < 0.01). Figure 4B shows that the pH value of Tri-HCL solutions in the Co (0, 1, 2.5, 5, 10) groups revealed a synchronously ascending state over the prolonged soaking time.

Figure 4C–E shows the surface morphology of the Co (0, 1, 2.5, 5, 10) scaffolds after mineralization in vitro. As displayed in Fig. 4D and E, the Co (0, 1, 2.5, 5) scaffolds showed apparent mineralization effects in vitro. A particulate and loose microstructure was consistently dispersed on the surface of the Co (0, 1, 2.5, 5) scaffolds, and cracks were observed. The XRD data (Fig. 4F) demonstrated that the mineralization phases of the Co (0, 1, 2.5, 5) scaffolds are hydroxyapatite. However, the Co10 scaffolds showed none of the changes mentioned above.

### Mechanical properties of the Co (0, 1, 2.5, 5, 10) scaffolds

The mechanical properties of the Co (0, 1, 2.5, 5, 10) scaffolds were obtained by compressive strength testing. As shown in Fig. 4G, the compressive strength of the Co (1, 2.5, 5) scaffolds slightly decreased with increasing Co^2+^ ion content compared with the Co0 scaffolds; remarkably, the compressive strength of the Co10 scaffold sharply declined, and the differences between the Co10 group and the other groups were significant (*p* < 0.01).

### Biocompatibility of the Co (0, 1, 2.5, 5, 10) scaffolds

**Fig. 5:**
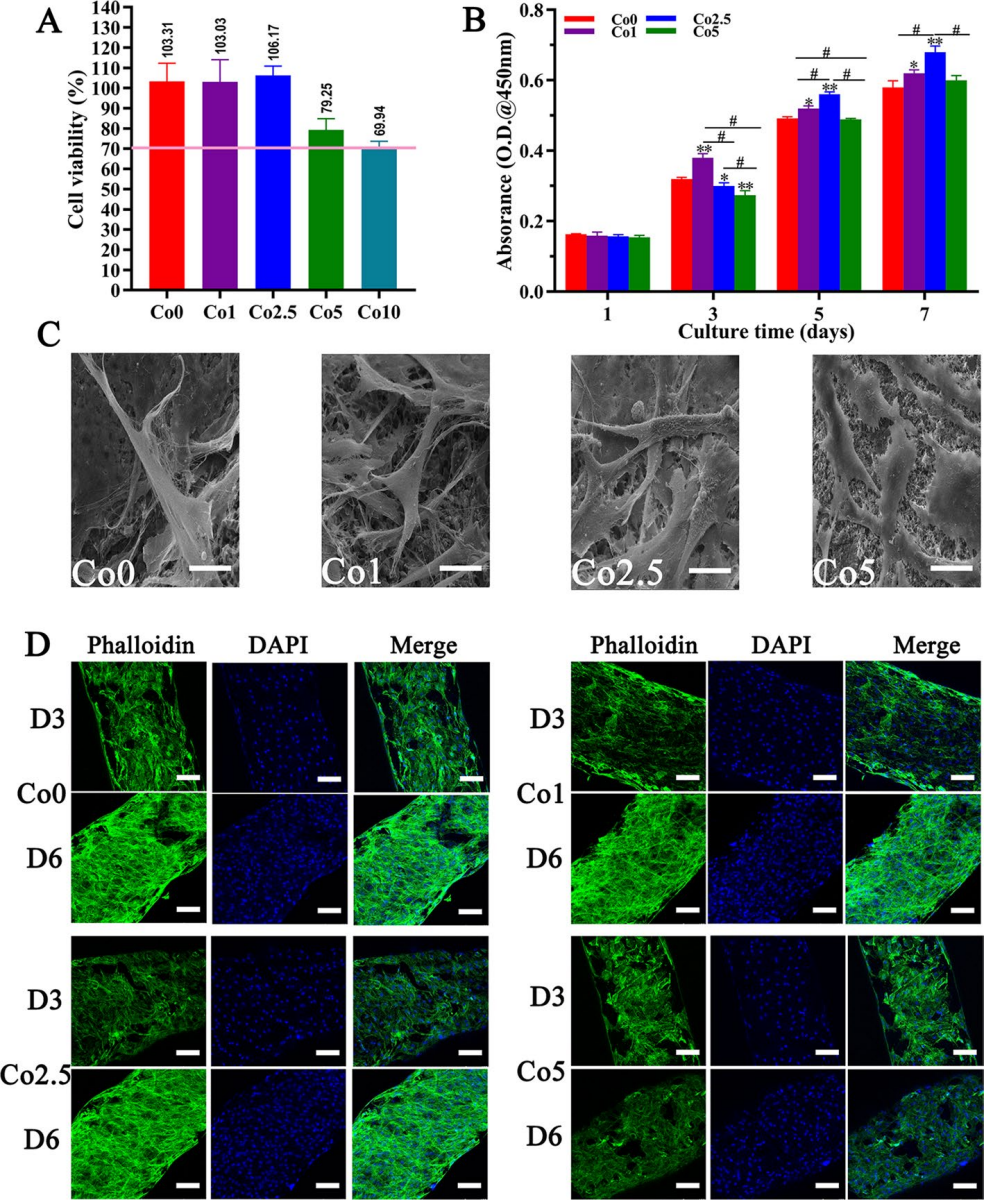
Biocompatibility of the Co (0, 1, 2.5, 5, 10) scaffolds. **A** Cytotoxicity analysis of the Co (0, 1, 2.5, 5, 10) scaffolds; less than 70% cell viability was considered cytotoxic. **B** The proliferation of rBMSCs cultured on the Co (0, 1, 2.5, 5) scaffolds. The data are expressed as the mean ± SD. *p < 0.05 and **p < 0.01, comparison with the Co0 group; ^#^*p* < 0.05, comparison among the Co (1, 2.5, 5) groups. **C** SEM micrographs of rBMSCs morphology cultured on Co (0, 1, 2.5, 5) scaffolds for 3 days (scale bars = 10 µm). **D** CLSM immunofluorescence micrographs of rBMSCs morphology cultured on the Co (0, 1, 2.5, 5) scaffolds for 3 days and 6 days. Cell skeleton staining with phalloidin (green). Nuclear staining with DAPI (blue) (scale bars = 100 µm)

Preliminary evaluation of the biocompatibility on the Co (0, 1, 2.5, 5, 10) scaffolds was detected by means of a cytotoxicity assay for subsequent biological studies. As shown in Fig. 5A, the cell viability in the Co (0, 1, 2.5, 5) groups was 103.31%, 103.03%, 106.17%, and 79.25%, which were all greater than 70% and did not show obvious cytotoxicity, while the cell viability in the Co10 group was 69.94%, which was less than 70% and displayed obvious cytotoxicity.

To further assess the biocompatibility of the Co (0, 1, 2.5, 5) scaffolds, the proliferation of rat BMSCs (rBMSCs) on the Co (0, 1, 2.5, 5) scaffolds was measured. As shown in Fig. 5B, the OD values in the Co (0, 1, 2.5, 5) groups increased with the extension of culture time, which indicated that rBMSCs cultured on the Co (0, 1, 2.5, 5) scaffolds proliferated well. In particular, after 24 h of incubation, there were no significant differences in cell proliferation among the Co (0, 1, 2.5, 5) scaffolds. At day 3, the proliferation of rBMSCs on the Co1 scaffold showed a significant difference from that on the Co (0, 2.5, 5) scaffolds, whereas cell proliferation on the Co (2.5, 5) scaffolds was less than that on Co0 scaffold. Interestingly, as the culture time increased, the Co2.5 scaffold effectively promoted rBMSCs proliferation at days 5 and 7 compared with the Co (0, 1, 5) scaffolds. However, the Co5 scaffold showed a slight decrease in cell proliferation compared with the other Co-doped scaffolds at each time point.

The adhesion and morphology of rBMSCs on the Co (0, 1, 2.5, 5) scaffolds were also observed. Figure 5C and D shows SEM images of rBMSCs at day 3 and confocal laser scanning microscope (CLSM) images of rBMSCs at days 3 and 6 after seeding on the Co (0, 1, 2.5, 5) scaffolds, respectively. As revealed by the SEM images in Fig. 5C, the rBMSCs on Co the (0, 1, 2.5) scaffolds attached and spread well with more abundant filopodia than those on the Co5 scaffold. No obvious distinction was noted in cell morphology on the Co (0, 1, 2.5) scaffold surfaces. Intriguingly, the CLSM results presented a similar trend as the Cell Counting Kit-8 (CCK-8) assay data and SEM images. In the CLSM results, cell skeleton was stained green, and the nucleus was stained blue. As shown in Fig. 5D, there were few rBMSCs on the surface of the Co (0, 1, 2.5, 5) scaffolds at day 3, and there were more rBMSCs at day 6, as observed by CLSM images. However, compared with the Co (0, 1, 2.5) scaffolds, the Co5 scaffold had slightly fewer cells at each time point.

### In vitro* angiogenic properties of the Co (0, 1, 2.5, 5) scaffolds*

**Fig. 6:**
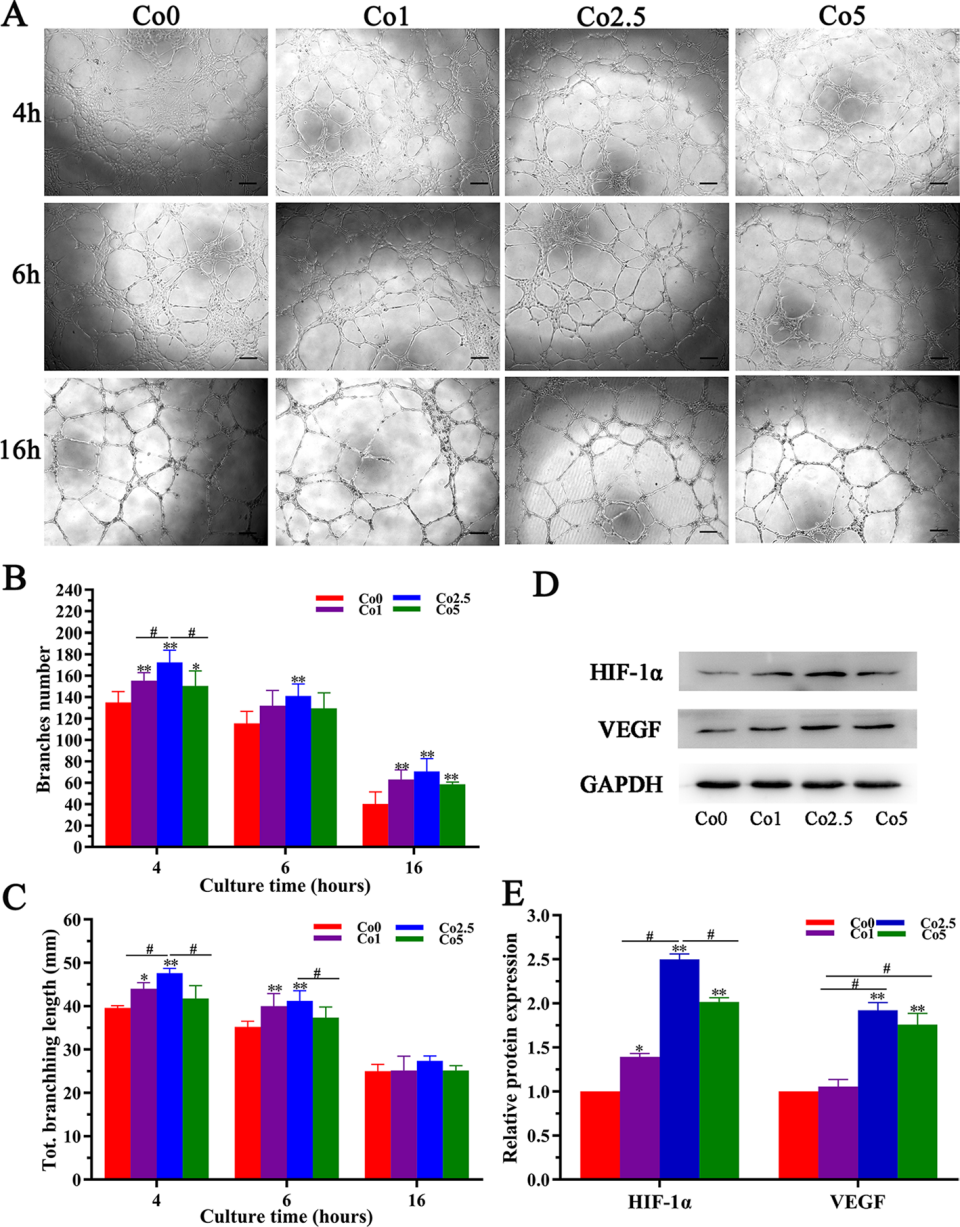
In vitro angiogenic property of the Co (0, 1, 2.5, 5) scaffolds. **A** Micrographs of HUVECs cultured in Co (0, 1, 2.5, 5) extracts for 4, 6, and 16 h (scale bar = 100 µm). **B**, **C** Statistical analysis of the branches number and total branching length. **D** Western blot analysis of HIF-1α and VEGF proteins in rBMSCs cultured in Co (0, 1, 2.5, 5) extracts for 7 days and their quantitative analysis (**E**). The data are expressed as the mean ± SD. *p < 0.05 and **p < 0.01, comparison with the Co0 group; ^**#**^*p* < 0.05, comparison among the Co (1, 2.5, 5) groups

The angiogenic properties of the Co (0, 1, 2.5, 5) scaffolds were investigated by conducting an in vitro tubule formation assay. As shown in Fig. 6A, human umbilical vein endothelial cells (HUVECs) in all groups sprouted and self-assembled to form branched nodes and circles after 4 h of culture, finally forming continuous tubular networks. It is worth noting that the Co2.5 group induced the most tube-like structures among all of the groups. After 6 h of culture, the Co2.5 group maintained its advantage in stimulating tubule formation. Although the HUVECs began to undergo apoptosis after 16 h of culture, which was the case in all of the groups, the cells in the Co2.5 group displayed a better network structure than the cells in Co the (0, 1, 5) groups. Quantitative analysis of the branches number (Fig. 6B) and total branching length (Fig. 6C) showed significantly higher values in Co (1, 2.5, 5) groups than that in the Co0 groups, especially in the Co2.5 group.

HIF-1α and VEGF expression in rBMSCs incubated with Co (0, 1, 2.5, 5) extracts was also analyzed. As exhibited in Fig. 6D and E, both HIF-1α and VEGF expression was significantly upregulated in the Co (1, 2.5, 5) groups compared with that of the Co0 group. In particular, the Co2.5 group showed greatly enhanced expression of HIF-1α and VEGF in comparison with that of the Co 1 and Co 5 groups.

### In vitro* osteogenic properties of the Co (0, 1, 2.5, 5) scaffolds*

**Fig. 7:**
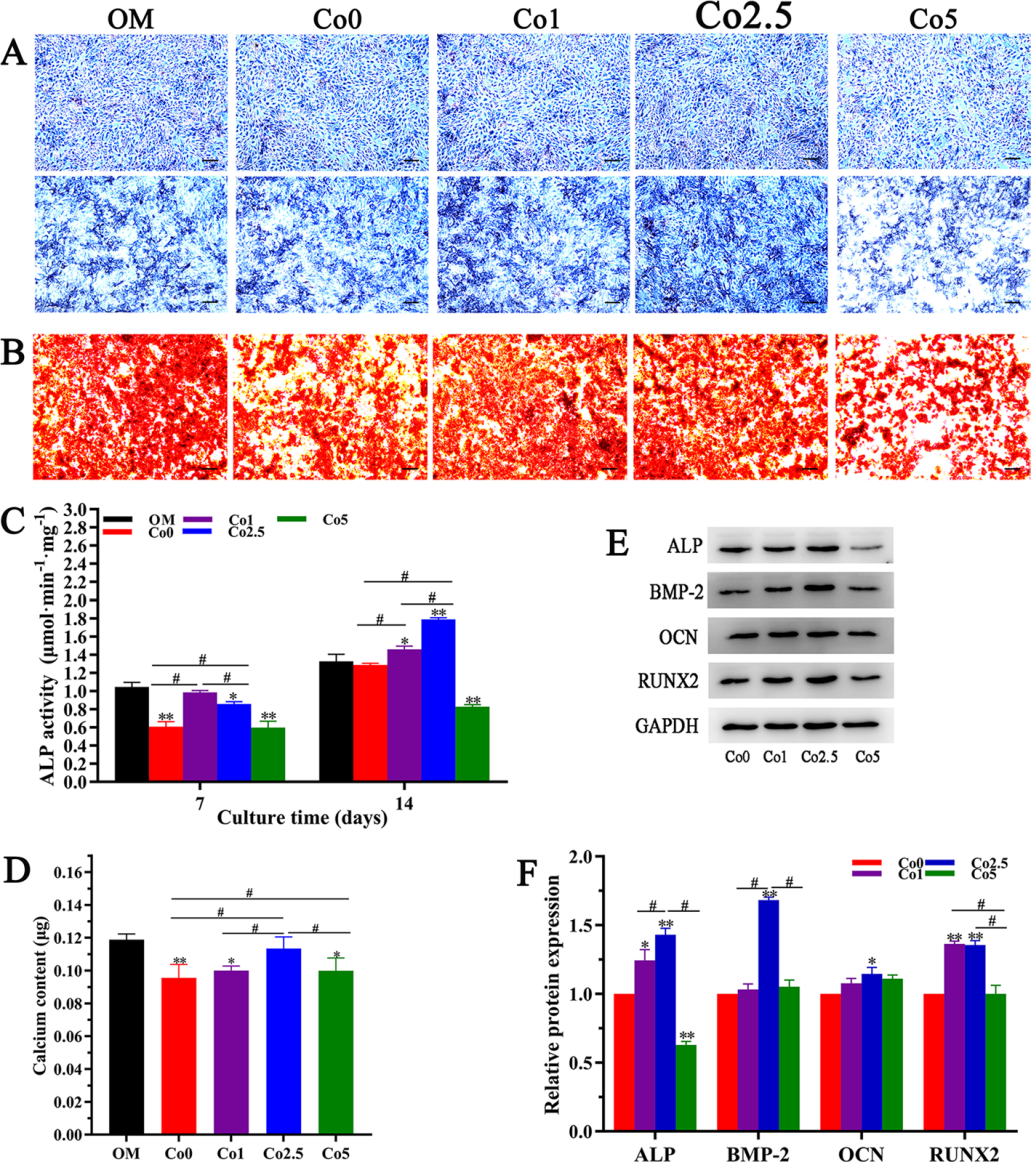
In vitro osteogenic properties of the Co (0, 1, 2.5, 5) scaffolds. **A** Micrographs of ALP staining at days 7 and 14 and **B** alizarin red staining at day 21 are shown (scale bars = 100 µm). The quantitative analysis of ALP activity (**C**) and calcium content (**D**) are shown. The group in which rBMSCs were cultured in osteogenic medium (OM) was set as the positive control group. The data are expressed as the mean ± SD. *p < 0.05 and **p < 0.01, comparison with the OM group; ^**#**^*p* < 0.05, comparison among the Co (0, 1, 2.5, 5) groups. **E** Western blot analysis of osteogenic markers (ALP, BMP-2, OCN and RUNX2) and their quantitative analysis (F) in rBMSCs stimulated by the Co (0, 1, 2.5, 5) extracts for 14 days. The data are expressed as the mean ± SD. *p < 0.05 and **p < 0.01, comparison with the Co0 group; ^**#**^*p* < 0.05, comparison among the Co (1, 2.5, 5) groups

The osteogenic properties of the Co (0, 1, 2.5, 5) scaffolds were measured by alkaline phosphatase (ALP) staining and alizarin red staining. As shown in Fig. 7A, after culture for 7 days, the intensity of ALP staining in the Co (0, 1, 2.5, 5) groups was similar to that in the osteogenic medium (OM) group. With prolonged culture time, the staining intensity in all groups on day 14 was higher than that on day 7. Moreover, the Co2.5 group showed more intense staining than the other groups. With the exception of the initial osteogenic marker, the terminal state of osteogenic differentiation of rBMSCs was examined via alizarin red staining. As displayed in Fig. 7B, alizarin red staining showed that a large number of red-stained calcium nodules were formed in all groups, and the calcium nodule numbers in the Co (1, 2.5) groups and OM group were significantly higher than those in the Co (0, 5) groups. To quantitatively evaluate the osteogenic properties of the Co (0, 1, 2.5, 5) scaffolds, the ALP activity and calcium content were analyzed. As revealed in Fig. 7C, the ALP activity in the Co (0, 1, 2.5, 5) groups was lower than that in the OM group over 7 days of culture. Additionally, the Co1 group enhanced ALP activity among the Co (0, 1, 2.5, 5) groups. Interestingly, the Co2.5 group exhibited markedly increased ALP activity compared to with the other groups over 14 days of stimulation, the difference was significant (*p* < 0.05). As shown in Fig. 7D, the group exposed to the Co2.5 extract had higher calcium contents than the groups exposed to the Co (0, 1, 5) extracts. Although the OM group had a higher calcium content, the differences between the Co2.5 and OM groups were not significant (*p* > 0.05).

The expression levels of osteogenic markers, including ALP, bone morphogenetic protein-2 (BMP-2), runt-related transcription factor 2 (RUNX2), and osteocalcin (OCN), in rBMSCs cultured in Co (0, 1, 2.5, 5) extracts at day 14 were also examined. As shown in Fig. 7E and F, the Co2.5 group exhibited a more significant upregulation of the expression of ALP and BMP-2 than the Co (0, 1, 5) groups at day 14. Although OCN expression did not show a significant difference among the Co (1, 2.5, 5) groups, OCN expression in the Co2.5 group was higher than that in the Co0 group. RUNX2 expression in the Co2.5 group at day 14 was also higher than that in the Co (0, 5) groups but slightly lower than that in the Co1 group, which showed no noticeable difference (*p* > 0.05).

### In vivo* bone regeneration of the Co2.5 scaffold*

A cranium bone defect model with 5 mm in diameter in rats was constructed to validate the in vivo osteogenic properties of Co-doped scaffolds. Since the in vitro results revealed that the osteogenic and angiogenic properties of the Co2.5 scaffold were superior to those of the Co (0, 1, 5) scaffolds, the Co2.5 scaffold was applied to investigate whether it resulted in enhanced bone regeneration in vivo.

**Fig. 8:**
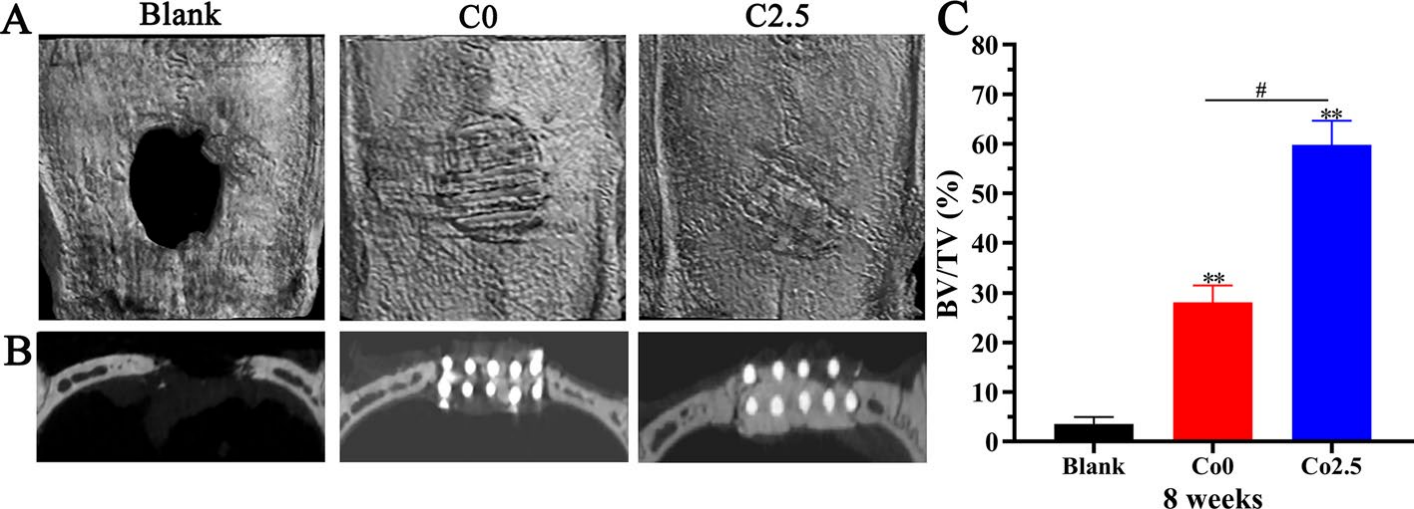
**A** Micro-CT images of cranium defects implanted with Co0 and Co2.5 scaffolds as well as the blank group. **B** The planar images of the three groups. **C** BV/TV analysis. The data are expressed as the mean ± SD. *p < 0.05 and **p < 0.01, comparison with the blank group; ^**#**^*p* < 0.05, comparison between the Co0 and Co2.5 groups

At 8 weeks after implantation, CT-constructed images were obtained to evaluate the extent of new bone formation by micro-CT. As shown in Fig. 8A, the round bone defect in the blank group clearly existed, and a small amount of bone-like tissue could be observed on the edge of the defect. In the Co0 group, scaffolds in bone defects were visible in situ, and the edge of the scaffold was covered by bone-like tissue. Notably, the scaffold in the Co2.5 group was almost integrated with the surrounding normal bone tissue and was not easily visible, and the scaffold–host bone interface was completely fused. In addition, as displayed in planar views (Fig. 8B), a larger amount of new bone along the margin and in the center of the defect was formed in the Co2.5 group compared with the Co0 and blank groups. Quantitative evaluation of new bone formation was performed using a bone volume/tissue volume (BV/TV) assay. As shown in Fig. 8C, the BV/TV was the highest in the Co2.5 group, followed by the Co0 group and blank group. Compared with that in the blank and Co0 groups, the BV/TV in the Co2.5 group was significantly increased (*p* < 0.01).

**Fig. 9:**
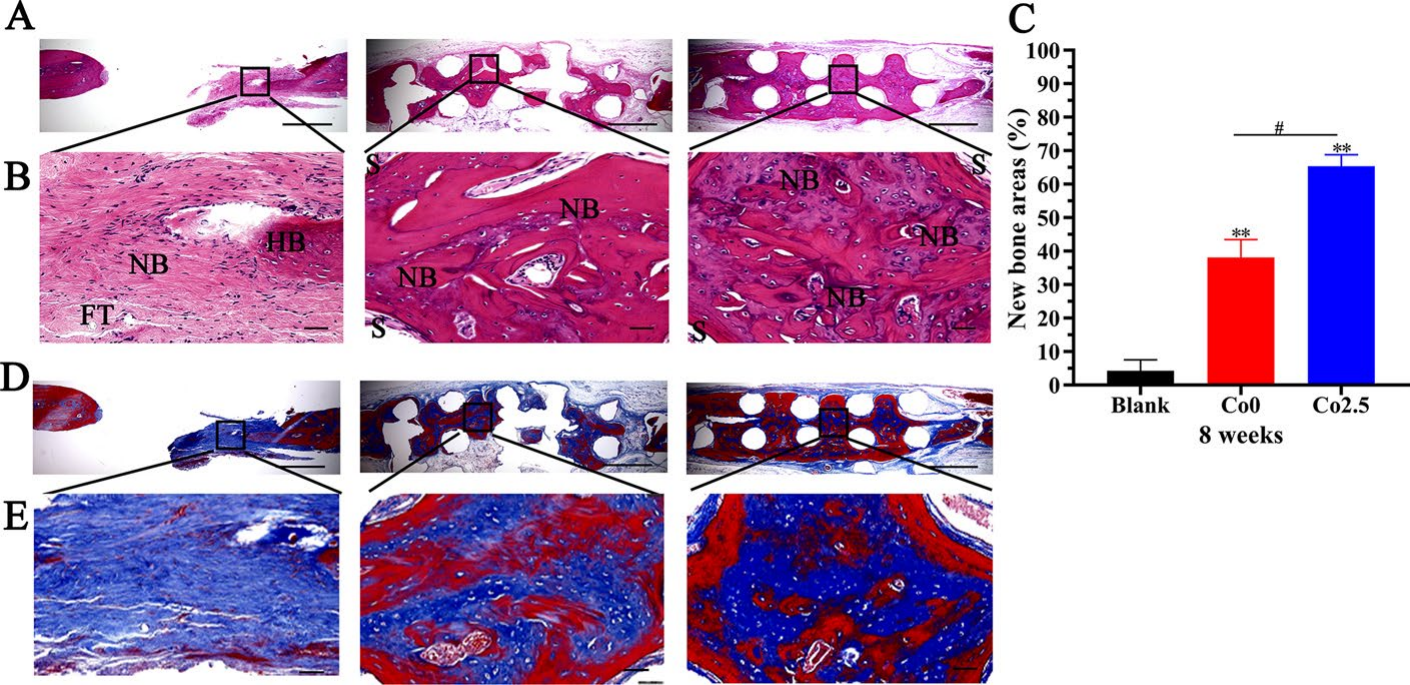
**A**, **B** H&E staining images of three groups (scale bar: Figure A = 50 µm, Figure B = 100 µm, NB: new bone, HB: host bone, FT: fibrous tissue, S: scaffolds). **C** New bone areas were calculated by Image-Pro Plus 6.0 software. The data are expressed as the mean ± SD. *p < 0.05 and **p < 0.01, comparison with the blank group; ^**#**^*p* < 0.05, comparison between the Co0 and Co2.5 groups. **D**, **E** Masson’s trichrome staining images (scale bar: Figure **D** = 50 µm, Figure **E** = 100 µm)

Following micro-CT analysis, the samples were performed histologically to assess new bone formation. The hematoxylin and eosin (H&E) staining data indicated a bone regeneration tendency parallel to the above micro-CT data. New bone tissue was stained with different shades of red in H&E staining. As revealed in Fig. 9A and B, new bone tissue was found in the center and along with the margin of the defect in the Co2.5 group, but was relatively rare in the Co0 group, with the least new bone formation in the blank group. Furthermore, the new bone area was evaluated quantitatively. As displayed in Fig. 9C, the new bone area was significantly increased in Co2.5 group, followed by the Co0 and blank groups, and the differences among groups were significant (*p* < 0.01). Additionally, Masson’s trichrome staining was implemented to histologically evaluate the maturity of new bone tissue. Masson’s trichrome staining showed that collagen fibers and immature bone tissue were blue, and mature bone tissue was red. As shown by Masson’s trichrome staining in Fig. 9D and E, in the blank group, only a small amount of collagen fibers and immature bone tissue were formed at the defect edge. Although the new bone tissue formed in the Co2.5 group and Co0 group was mainly mature bone tissue, the amount of new bone tissue in the Co2.5 group was significantly greater than that in the Co0 group.

## Discussion

The doping of inorganic ions into bioceramics is an effective strategy for modification, and has attracted extensive attention as a burgeoning avenue for promoting bone regeneration [22, 23]. In the present study, Co-doped scaffolds were synthesized by the solid-phase sintering method and SE-3DP. A color difference was observed between the Co-doped and -undoped scaffolds. The color of the Co0 scaffold was white, whereas the Co (1, 2.5, 5, 10) scaffolds turned purple with increasing Co-doping amount. Color variation provided a clue that Co^2+^ ions were successfully doped into CLP. Doping was investigated with the purpose of replacing Ca^2+^ ions from the CLP with Co^2+^ ions. Ion doping can cause variations in the microstructure and crystal morphology of bioceramics depending on the property, size, and amount of the dopant [24]. The XRD patterns of the Co (1, 2.5, 5, 10) scaffolds were almost identical to that of the Co0 scaffold, and there was no additional peak corresponding to Co, which indicated that Co^2+^ ions might have been completely doped into the CLP structure. With increasing Co-doping content, the movement of the XRD profile toward a slight right shift might be attributed to the substitution of the low ionic radius Co^2+^ (0.070 nm) for the high ionic radius of Ca^2+^ (0.099 nm), which may cause the contraction of the crystal lattice [16, 25, 26]. The ionic radius mismatch of the doped ions mentioned above may also explain why the crystal planes (110), (128), (4,0,10), and (2,0,20) changed when the doping percentage of Co^2+^ ions increased up to 1 mol%. The variation in crystal planes is usually closely related to a reduction in crystallinity and crystal size. Hence, it can be concluded that partial replacement of Ca^2+^ ions with Co^2+^ ions may lead to smaller crystal sizes and reduced crystallinity, which can account for the changes in crystal size on Co the (0, 1, 2.5, 5, 10) scaffolds’ surface. Similarly, the Raman spectra of the Co (1, 2.5, 5, 10) scaffolds also showed that Co doping may result in a decrease in the lattice constant of CLP [27]. Together, Co doping has a certain influence on CLP structure, and the greater the doping amount is, the greater the influence, especially as the Co-doping concentration increased to 1 mol%.

The degradation and resorption of artificial bone grafts play a significant role in the bone repair process [28], which occurs in parallel with osteogenesis and bone regeneration. As artificial bone grafts are degraded, new bone tissue grows into bone defect sites to complete bone repair. The data in Fig. 4A demonstrate that Co-doped CLP possessed excellent degradation properties compared with Co undoped CLP, which was beneficial for accelerating bone formation. Additionally, the mineralization potential of artificial bone grafts contributes to the enhanced bone-bonding process. The mineralization results showed that the Co (1, 2.5, 5) scaffolds had mineralization effects similar to those of the Co0 scaffold, which was conducive to firm bone binding to host bone after implantation in vivo. However, the Co10 scaffold showed no mineralization properties, which may be attributed to distortion of the crystal lattice caused by partial replacement of Ca^2+^ ions with Co^2+^ ions.

A higher compressive strength presents a higher fracture toughness [29]. Although the compressive strength of the Co (1, 2.5, 5, 10) scaffolds decreased with increasing Co^2+^ ion content in comparison with the Co0 scaffold, especially the Co10 scaffold, these data with respect to the Co (1, 2.5, 5) scaffolds remained in the range of human trabecular bone [30], suggesting that the Co (1, 2.5, 5) scaffolds could also bear external stress and resist fracture. The possible reason accounting for the low mechanical properties of the Co10 scaffold is that doping with Co^2+^ ions lead to increased lattice distortion of CLP, resulting in large penetrating cracks. Additionally, a desired artificial bone graft should an exhibit an interconnected porous structure, in which the pore diameters should be between 200 and 500 μm, and the porosity should be above 50% [31, 32]. An ideal interconnected porous structure not only maintains certain mechanical properties, but also favors cell penetration, angiogenesis, and osteogenesis [31, 32]. In this work, the interconnected pore size was approximately 400 μm × 400 μm, and the porosity was 40% (Fig. 3A), slightly less than the designated porosity due to printing precision issues and the sintering process. However, the ideal scaffold must compromise and balance porosity and compressive strength. In general, the doping of Co^2+^ ions have a certain influence on the compressive strength, degradation, and mineralization properties of CLP, and the greater the doping amount is, the greater the influence.

As degradation proceeds, the Co-doped scaffolds could release Co^2+^ ions into the surrounding environment; the amount released mainly depends on the concentration of Co incorporated into the scaffolds. Related reports have confirmed that Co^2+^ ions at high concentrations may cause cell and tissue damage [33]. With this in mind, we preliminarily detected the cytotoxicity of the Co (0, 1, 2.5, 5, 10) scaffolds for subsequent biological studies. As shown in Fig. 5A, the Co (0, 1, 2.5, 5) scaffolds did not show obvious cytotoxicity (cell viability > 70%), while the Co10 scaffold displayed obvious cytotoxicity (cell viability < 70%). The reason for cytotoxicity on the Co10 scaffold may be that doping Co has a significant influence on the degradation performance of CLP, and the greater the doping amount, the more obvious the degradation, especially the Co10 scaffold. As the degradation of the Co10 scaffold increases, the amount of released ions increases accordingly. According to the results of the characterization, physicochemical properties, compressive strength, and cytotoxicity assay, we selected the Co (0, 1, 2.5, 5) scaffolds for subsequent in vitro research.

Although the Co (0, 1, 2.5, 5) scaffolds showed no significant cytotoxicity, it was necessary to further assess the biocompatibility of the Co (0, 1, 2.5, 5) scaffolds. It is vital that osteoprogenitors proliferate well on artificial bone grafts in bone regeneration and bone remodeling processes [34]. Therefore, we cultured rBMSCs on Co (0, 1, 2.5, 5) scaffolds and measured cell proliferation. The CCK-8 results demonstrated that rBMSCs could proliferate well on the Co (0, 1, 2.5, 5) scaffolds over the culture time, particularly on the Co2.5 scaffold, showing favorable biocompatibility. The prerequisite that artificial bone grafts exert biological functions is that rBMSCs can easily adhere and spread on the surface of artificial bone grafts. As far as the artificial bone graft is concerned, its components and surface morphology will have a certain impact on cell adhesion. SEM images revealed that rBMSCs were healthy spread and attached well on the surface of the Co (0, 1, 2.5) scaffolds with abundant filopodia compared with the Co5 scaffold. Intriguingly, the CLSM results presented a similar trend as the CCK-8 assay data and SEM images, i.e., rBMSCs could attach and proliferate well on the Co (0, 1, 2.5, 5) scaffolds over the culture time. However, the Co5 scaffold showed a slight decrease in cell proliferation compared with the other Co-doped scaffolds at each time point, which may be attributed to the fact that although the cell viability of the Co5 scaffold was slightly greater than the critical value of 70% (cell viability 79% > 70%) and showed no obvious cytotoxicity, it may still had a certain influence on the metabolic state of rBMSCs. In summary, all of these results clearly confirmed that, among the Co (0, 1, 2.5, 5) scaffolds, the Co2.5 scaffold possesses excellent biocompatibility, which is one of the requirements of artificial bone grafts.

The principal reason for incorporating Co^2+^ ions in artificial bone grafts is dependent on the inherent property of Co^2+^ ions to accelerate angiogenesis [35]. Co^2+^ ions, a chemical inducer, can deactivate PHD, improving the HIF-1α concentration and therefore imitating hypoxic conditions under normal oxygen tension [36]. In turn, HIF-1α can upregulate the expression of angiogenic genes such as VEGF, which have close relationships with bone tissue development and regeneration [37]. In addition, VEGF, as a key regulator of the growth of the vascular network, can affect endothelial cells by stimulating their migration, proliferation, and eventually the formation of tubular structures [38]. Nevertheless, studies remain rare regarding whether incorporating Co into CLP can also induce a hypoxic response to promote angiogenesis, and thus enhance bone regeneration. Our results showed that HUVECs in the Co (1, 2.5, 5) groups displayed a better network structure than those in the Co0 group, demonstrating that Co^2+^ ions released from Co (1, 2.5, 5) scaffolds are beneficial to the formation of tube-like structures in HUVECs, especially in the Co2.5 group. Moreover, the Co2.5 group showed greatly enhanced expression of HIF-1α and VEGF compared with that of the Co1 and Co5 groups, while the Co0 group showed no obvious effect on the expression of HIF-1α and VEGF. These results indicated that the Co-doped CLP can simulate a hypoxic environment under normal oxygen pressure and thus promote VEGF expression to accelerate angiogenesis. Since the Co (0, 1, 2.5, 5) scaffolds have similar substrates, it is suggested that the release of Co^2+^ ions from the Co (1, 2.5, 5) scaffolds mainly contributes to the promotion of HIF-1α and VEGF protein expression. Therefore, the incorporation of Co^2+^ ions into CLP is an available way to induce the hypoxic cascade to enhance angiogenesis for bone regeneration. Together with tubule formation data, the Co2.5 extract may be more conducive to angiogenesis than the Co (0, 1, 5) extracts.

An ideal artificial bone graft should possess excellent osteogenic properties to accelerate bone repair. Acting as an extracellular matrix, the components of artificial bone grafts, including inorganics and organics, play a vital role in promoting osteogenic differentiation of osteoprogenitors. Hence, we qualitatively and quantitatively assessed the influence of the Co (0, 1, 2.5, 5) scaffolds on the osteogenic differentiation of rBMSCs in terms of initial and terminal osteogenic markers. The results indicated that the Co (0, 1, 2.5, 5) scaffolds could promote osteogenic differentiation of rBMSCs, and the osteogenic performance of Co2.5 scaffold was superior to that of the Co (0, 1, 5) scaffolds.

The transitional stages of osteoprogenitors into osteoblasts involve the spatiotemporal expression of osteogenic genes, e.g., Runx2 and OCN [39]. Therefore, the effect of the Co (0, 1, 2.5, 5) scaffolds on the expression levels of osteogenic markers, including ALP, BMP-2, RUNX2, and OCN, in rBMSCs was analyzed. These data further indicated that the Co2.5 scaffold could promote osteogenic markers to higher expression levels than the Co (0, 1, 5) scaffolds. Some possible explanations for these findings are as follows. First, the stabilization of HIF-1a activated by Co^2+^ ions can induce the expression of a number of genes that are activated in fracture repair [40]. Second, Co^2+^ ions could also act as an agonist of Ca^2+^ ions and induce Wnt5 signal amplification, improving the expression of osteogenesis-related genes [41]. However, the expression of the aforementioned osteogenic genes did not increase with increasing Co-doping percentage. The reason might be the possibility of an antagonistic effect of Co^2+^ ions on Ca^2+^ ions at a high Co concentration and a reduction in expression [41].

Bone defect repair by artificial bone grafts involves a series of complex processes, and therefore, it is necessary to verify the bone regeneration of artificial bone grafts in vivo. Since the in vitro experiments demonstrated that the Co2.5 scaffolds showed excellent osteogenic and angiogenic properties compared with other scaffolds, the Co2.5 scaffold was employed to observe whether it also resulted in enhanced in vivo bone regeneration. The in vivo results demonstrated no significant new bone tissue formation in the blank group, indicating that the critical-sized defect model was suitable for evaluating the osteogenic capacity of scaffolds in vivo. Furthermore, the Co2.5 scaffold could significantly promote bone regeneration and had a positive effect on bone defect repair in comparison with the Co0 scaffold. It has been widely accepted that the local hypoxic environment is indispensable for bone regeneration. Since the Co2.5 and Co0 scaffolds are identical in the substrates and fabrication techniques, the sole discrepancy between the two scaffolds was the incorporation of Co^2+^ ions. Co^2+^ ions released from the Co2.5 scaffold can mimic hypoxic conditions by stabilizing HIF-1α activity. Hypoxia plays an essential role in improving angiogenesis and osteogenesis by inducing the HIF-1α pathway and promoting the migration, proliferation, differentiation, and VEGF secretion of MSCs [42, 43], thus promoting bone regeneration. On the other hand, the enhanced expression of VEGF, as one of the downstream regulators of HIF-1α, facilitates the angiogenic process and neovascularization of scaffolds. Taken together, the above findings suggested that adding Co^2+^ ions into CLP could improve bone regeneration by enhancing angiogenic and osteogenic capacities and is beneficial to bone defect repair.

However, there is another aspect to consider: most of the scaffolds appeared to remain after in vivo implantation for 8 weeks; in other words, the scaffolds showed limited signs of degradation despite undergoing a certain level of degradation. This finding was probably due to the short degradation time. Neo-bone formation could only occur through the surface and pores of scaffold within a limited time, while scaffold mostly remained without being replaced by neo-bone tissues. Although the Co2.5 scaffold partially degraded, the bone regeneration quality was excellent, demonstrating excellent bone defect repair properties. With respect to degradation, in-depth research may be required to optimize the scaffolds in the future. In short, this study showed that incorporating Co into CLP is an effective avenue to enhance the angiogenic and osteogenic properties of CLP. The Co2.5 scaffold is promising for use as an artificial bone graft with potentially improved bone regeneration.

## Conclusion

In this study, porous scaffolds were fabricated by doping CLP with various concentrations of Co (0, 0.1, 0.25, 0.5, 1 mol%) and SE-3D printing techniques, named Co0, Co1, Co2.5, Co5, and Co10. The incorporation of Co impacted the microstructure and physicochemical properties of CLP. The effect was enhanced along with increasing concentrations of Co doping and was especially evident in the Co10 scaffold. The in vitro data demonstrated that the Co (1, 2.5, 5) scaffolds showed excellent biocompatibility and osteogenic and angiogenic properties compared with the Co0 scaffold, especially the Co2.5 scaffold. In comparison with the Co0 scaffold, the Co2.5 scaffold significantly enhanced bone regeneration in rat calvarium defects at 8 weeks post-implantation. Together, our results suggest that the incorporation of Co into CLP is an available path to promote potential osteogenic and angiogenic properties. The Co2.5 scaffold holds promise for use as an artificial bone graft in bone repair with enhanced osteogenic and angiogenic potential.

## Materials and methods

### Materials

Polyvinyl alcohol, (NH_4_)_2_HPO_4_ (99%), Ca(NO_3_)_2_•4H_2_O (99%), Li3PO4 (99%), CoCl_2_ (99%), NH3•H2O (25–28%), and Tris–HCl were purchased from Sinopharm Chemical Reagent Co., Ltd. Simulated body fluid (SBF) was purchased from Leagene (China). Minimum Eagle's medium (MEM), Dulbecco’s modified Eagle’s medium/Ham’s F-12 (DMEM/F-12), and fetal bovine serum (FBS) were purchased from HyClone (USA). Endothelial cell medium was obtained from ScienCell (USA). The NBT/BCIP staining kit and detection kit for ALP activity and calcium content were purchased from Beyotime Company (China). The CCK-8 assay was obtained from Dojindo (Japan). MTT, phalloidin, and 4',6-diamidino-2-phenylindole (DAPI) were purchased from AAT Bioquest (USA). Primary antibodies against ALP, BMP-2, RUNX2, OCN, HIF-1α, VEGF, GAPDH, and HRP-conjugated secondary antibodies were obtained from Affinity (China).

### ***Synthesis of Ca***_***(10-x)***_***Co***_***x***_***Li(PO***_***4***_***)***_***7***_*** powders (x*** = ***0, 0.1, 0.25, 0.50, 1)***

Crystalline Ca_10_Li(PO_4_)_7_ (CLP), in which Ca^2+^ ions was substituted with different contents of Co^2+^ ions, was synthesized by a solid-phase sintering method with Ca(NO_3_)_2_•4H_2_O, (NH_4_)_2_HPO_4_, Li_3_PO_4_, and CoCl_2_ as precursors. The corresponding chemical formulation was Ca_(10-x)_Co_x_Li(PO_4_)_7_, in which x = 0, 0.1, 0.25, 0.5, and 1 mol%. Uniform synthesized powders were accordingly named Co1-CLP, Co2.5-CLP, Co5-CLP, and Co10-CLP. The CLP with no substitution of Co^2+^ ions for Ca^2+^ ions, named Co0-CLP, served as the control group and underwent a similar synthetic route. The details of synthetic route are described in S1.

### ***3D printing processes of Ca***_***(10-x)***_***Co***_***x***_***Li(PO***_***4***_***)***_***7***_*** scaffolds (x*** = ***0, 0.1, 0.25, 0.50, 1)***

Slurry extrusion 3D printing techniques were used to fabricate porous scaffolds. The slurry extrusion was controlled by the air pressure, which was performed under 0.3 to 0.5 MPa. To obtain optimal porous scaffolds, the speed extruding slurry was set as 30 to 40 mm/s. Porous scaffolds with 10 mm in diameter and 2 mm in thickness, abbreviated as Co0, Co1, Co2.5, Co5, and Co10 scaffolds, were obtained after being sintered at 950 °C for 3 h. The details of the printing processes are described in S2.

### Characterization of the Co (0, 1, 2.5, 5, 10) scaffolds

To characterize the Co (0, 1, 2.5, 5, 10) scaffolds, the XRD (Miniflex600, Rigaku), Raman spectrophotometer (LabRAM HR, HORIBA), and SEM (SU-8010, HITACHI) were performed to detect crystal phase, surface morphology, and crystal microstructure, respectively.

### Compressive strength of the Co (0, 1, 2.5, 5, 10) scaffolds

A testing machine (CMT4304, SANS) was applied to test the compressive strength of the Co (0, 1, 2.5, 5, 10) scaffolds (10 mm in diameter and 15 mm in thickness), and a constant displacement rate was set as 0.2 mm/min according to Chinese Standard GB/T 8489–2006 (*n* = 4).

### *Mineralization of the Co (0, 1, 2.5, 5, 10) scaffolds *in vitro

SBF was applied to investigate the in vitro mineralization ability of the Co (0, 1, 2.5, 5, 10) scaffolds. In detail, the Co (0, 1, 2.5, 5, 10) scaffolds were immersed in SBF at 37 °C for 30 days at a ratio of 100 ml/g. The SBF solution was replaced each 3 days. After 30 days of immersion, the scaffolds were removed from the SBF solution, rinsed with distilled water 3 times and dried at 120 ℃ for 12 h. SEM (G2, Phenom) and XRD were applied to detect the hydroxyapatite phase on the scaffolds surface.

### *Degradation properties of the Co (0, 1, 2.5, 5, 10) scaffolds *in vitro

To identify the degradation properties, the Co (0, 1, 2.5, 5, 10) scaffolds (*n* = 4) were immersed in Tris–HCl solution at 37 °C at a ratio of 200 ml/g. After the predetermined time (1, 2, 3, and 4 weeks), a pH meter was used to test the pH values of the Tris–HCL solution. Then, the scaffolds were removed from the solution and dried at 120 ℃ for 12 h, and their final weights were precisely measured. Weight loss was represented as a percentage of the primary weight.

### Cytotoxicity assessment of the Co (0, 1, 2.5, 5, 10) scaffolds

The cytotoxicity of the Co (0, 1, 2.5, 5, 10) scaffolds was determined using the MTT assay in mouse fibroblast cells (L-929 cells) according to ISO 10,993–5 2016. To prepare extracts, the Co (0, 1, 2.5, 5, 10) scaffolds were soaked in MEM medium at a ratio of 200 mg/ml at 37 °C for 24 h. After incubating, the minimum eagle's medium (MEM) was centrifuged, and the resulting supernatant was filtered through a 0.22 μm strainer for sterilization.

The optical density (O.D.) of each well was determined using a microplate reader (Multiskan Ascent, Thermo Fisher Scientific) at 570 nm. To calculate the decrease in viability compared with the blank group, the following formula was used: Via. % = 100 × *OD*_570e_/*OD*_570b_ (where *OD*_570e_ is the average value of the measured O.D. of the 100% extracts, and *OD*_570b_ is the average value of the measured O.D. of the blanks, *n* = 6). If viability was decreased to lower than 70% of the blank, the sample was regarded as showing cytotoxic potential.

### Cell culture

rBMSCs were isolated and cultured according to the approaches described in the previous work [44]. In short, the bone marrow collected from the femurs and tibias of the rats under aseptic conditions was suspended and placed into tissue culture flasks containing Dulbecco’s modified eagle’s medium/ham’s F-12 (DMEM/F-12) supplemented with 10% (v/v) FBS and 1% (v/v) penicillin/streptomycin solution, and incubated in a 5% CO_2_ incubator at 37 °C. The culture medium was replaced every 3 days until the primary rBMSCs were 80% confluent. The floating hematopoietic cells were removed by multiple medium changes. Subculture was performed through trypsinization when rBMSCs reached 80% confluence. Only early passage (p3–6) cells were selected for subsequent cellular experiments.

HUVECs were obtained from iCell Bioscience (China) and cultured in endothelial cell medium (ECM) containing 10% (v/v) FBS and 1% (v/v) penicillin/streptomycin solution.

### Biocompatibility of the Co (0, 1, 2.5, 5) scaffolds

#### The proliferation of rBMSCs cultured on the Co (0, 1, 2.5, 5) scaffolds

The proliferation of rBMSCs seeded on the Co (0, 1, 2.5, 5) scaffolds was evaluated using the CCK-8 assay according to the manufacturer's protocol. Briefly, the Co (0, 1, 2.5, 5) scaffolds (8 mm in diameter and 2 mm in thickness) were first transferred into 48-well plates. Then, rBMSCs were collected and resuspended at 5 × 10^4^ cells/ml. One hundred microliters of cell suspension was pipetted into each scaffold of 48-well plates and cultured for 1, 3, 5, or 7 days. At each preset timepoint, 20 µl of CCK-8 solution was added to each well and incubated for 2 h at 37 °C. After incubation, a microplate reader (Thermo Fisher Scientific, USA) was applied to measure the O.D. at 450 nm in a new 96-well plates. Five data in each group were selected to calculate the average and the standard deviation.

#### Morphology of rBMSCs cultured on the Co (0, 1, 2.5, 5) scaffolds

The adhesion and morphology of rBMSCs on the Co (0, 1, 2.5, 5) scaffolds were captured by SEM and CLSM. After coculturing for 3 days, the scaffolds were rinsed in PBS and then fixed in 2.5% glutaraldehyde solution for 30 min. Then, the cellular scaffolds were dehydrated in an ethanol series and finally dried in a vacuum freeze drier for 3 h. The dehydrated cellular scaffolds were covered with gold, and the morphological characteristics of rBMSCs on the Co (0, 1, 2.5, 5) scaffolds were observed by SEM (FEI Q25, USA).

After 3 and 6 days of culture, the cellular scaffolds were also set in 2.5% glutaraldehyde solution to fix for 30 min and then infiltrated with a 0.2% Triton X-100 solution for 5 min after rinsing twice with PBS. The samples were then incubated with fluorescein isothiocyanate phalloidin for 1 h and DAPI for 15 min. The cells were visualized by CLSM (Leica TCS SP8, Germany).

### Tubule formation in vitro

To observe the effect of the Co (0, 1, 2.5, 5) scaffolds on the angiogenic process of HUVECs, we conducted an in vitro tubule formation experiment by coculturing HUVECs with Co (0, 1, 2.5, 5) extracts. First, the Co (0, 1, 2.5, 5) extracts were prepared by soaking the Co (0, 1, 2.5, 5) scaffolds in ECM at a ratio of 200 mg/ml at 37 °C for 24 h. Then, the ECM was centrifuged, and the resulting supernatant was filtered through a 0.22 μm strainer for sterilization. Briefly, a Matrigel matrix was coated into a 96-well plate for complete gelation for 1 h at 37 °C. HUVECs were seeded on the Matrigel matrix with Co (0, 1, 2.5, 5) scaffold extracts for 4, 6, and 16 h of culture. The fields of view per well were imaged by an optical microscope (Leica DMi8, Germany). Five images were analyzed for each group using ImageJ software to quantify total branching length and branches number, which are representative of the angiogenic process in HUVECs.

### The effect of the Co (0, 1, 2.5, 5) scaffolds on HIF-1α and VEGF expression in rBMSCs

To prepare extracts for rBMSCs culture, the Co (0, 1, 2.5, 5) scaffolds were soaked in DMEM/F-12 medium at a ratio of 200 mg/ml at 37 °C for 24 h. After incubation, the DMEM/F-12 medium was centrifuged, and the resulting supernatant was filtered through a 0.22 μm strainer for sterilization.

A western blot assay was performed to detect HIF-1α and VEGF expression levels. First, RIPA buffer was added to lyse cells to extract protein after rBMSCs were cultured in Co (0, 1, 2.5, 5) extracts for 7 days, and the protein concentration was quantitated using a BCA Protein Assay Kit. Subsequently, whole-cell lysates were obtained for western blot analysis. Briefly, the same quantity of lysate from the Co (0, 1, 2.5, 5) groups was electrophoresed on SDS-PAGE gels and subsequently transferred onto PVDF membranes. Then, the membranes were blocked in 5% non-fat milk for 2 h and incubated with primary antibodies against HIF-1α (1:1000), VEGF (1:1000), and GAPDH (1:2000) for 24 h at 4 °C. After rinsing with TBST, the membranes were incubated with HRP-conjugated secondary antibodies (1:3000) for 2 h. The band signals of proteins were visualized using enhanced chemiluminescence. The quantitation of band intensity was performed using ImageJ software.

### The effect of the Co (0, 1, 2.5, 5) scaffolds on the osteogenic differentiation of rBMSCs alkaline phosphatase (ALP) staining and activity

The preparation of Co (0, 1, 2.5, 5) extracts was the same as that described in Sect. 2.12. Briefly, rBMSCs were seeded in 12-well plates at a density of 1 × 10^5^ cells/well. After rBMSCs reached 80% confluence, the original medium was replaced by various scaffold extracts. After 7 and 14 days of stimulation, ALP staining and activity assays were performed using an NBT/BCIP staining kit and a detection kit for ALP activity according to a previous protocol. rBMSCs cultured in osteogenic medium were chosen as the positive control group. Five samples in each group were used in these experiments.

### Alizarin red staining and calcium content

After 21 days of stimulation, 4% paraformaldehyde was used to fix rBMSCs for 30 min at room temperature. After gentle washing with deionized water, alizarin red solution was added and incubated with rBMSCs for 30 min to visualize the calcium nodules. The calcium nodules were stained as red spots and imaged by light microscopy (Leica DMi8, Germany). To quantify the mineralization properties of different scaffolds, a detection kit for calcium content was utilized. A standard curve was constructed according to the manufacturer’s protocol, and from the equation of the trendline, the calcium content of each sample was calculated with the trendline equation. rBMSCs cultured in osteogenic medium were chosen as the positive control group. Five samples in each group were used in these experiments.

### The effect of the Co (0, 1, 2.5, 5) scaffolds on osteogenic protein expression in rBMSCs

Western blot assay was performed to investigate the efficacy of Co (0, 1, 2.5, 5) extracts on osteogenic protein expression. In brief, rBMSCs were incubated with Co (0, 1, 2.5, 5) extracts for 14 days and lysed in RIPA buffer to extract protein. Then, the protein concentration was determined using a BCA Protein Assay Kit. Subsequently, whole-cell lysates were obtained for western blot analysis. Briefly, the same quantity of proteins from Co (0, 1, 2.5, 5) groups was resolved on SDS-PAGE gels and subsequently transferred to PVDF membranes. Then, the membranes were blocked with 5% non-fat milk for 2 h and incubated with primary antibodies against ALP (1:1000), BMP-2 (1:1000), RUNX2 (1:1000), OCN (1:1000), and GAPDH (1:2000) for 24 h at 4 °C. The membranes were then incubated with HRP-conjugated secondary antibodies (1:3000) for 2 h at room temperature after washing with TBST. The band signals of proteins were detected by enhanced chemiluminescence. The quantification of band intensity was analyzed by ImageJ software.

### In vivo* bone defect repair*

The experimental protocols for all animals in this study were carried out according to the guidelines approved by the Ethics Committee at the First Affiliated Hospital of Sun Yat-sen University. Co0 and Co2.5 scaffolds with 5 mm in diameter and 2 mm in thickness were prepared and sterilized. Eighteen male Sprague–Dawley (SD) rats (Southern Medical University Experiment Animal Center, Guangzhou, China) aged 8 weeks were used and divided into the following three groups (*n* = 6): the blank group; the Co0 group; and the Co2.5 group.

### Animal surgery and scaffold implantation

Briefly, 5% chloral hydrate was managed via intraperitoneal injection for animal anesthesia. A critical bone defect with 5 mm in diameter in the parietal bone was created in SD rats by an electric trephine drill without injuring the underlying sagittal sinus and dura matter. The Co0 and Co2.5 scaffolds filled in the defect, while the blank group received no scaffolds. Then, absorbable sutures were used to stitch the tissue and skin layer by layer. After implantation, the animals were monitored daily by visual observation for signs of inflammation, food intake, activity, and any adverse reaction during the experimental time.

At 8 weeks after the operation, 18 rats were immolated by an overdose of chloral hydrate injected intraperitoneally, and the craniums including the bone defect site with surrounding bone tissue were collected for following analysis. The harvested rat braincase specimens were fixed 10% neutral-buffered formalin for 24 h at room temperature and subjected to micro-CT scanning and histological analysis.

### Micro-CT analysis

A micro-CT system (SkyScan 1276, Micro-CT, Bruker, Germany) was applied to scan each braincase, and NRecon software (Bruker, Germany) was used to reconstruct craniums. Then, CTvox software was utilized to perform 3D analyses. A cylindrical region of interest (ROI) with 5 mm in diameter was outlined at the center of the single defect, completely enclosing the new bone in the defect site. The bone volume (BV; mm^3^) and tissue volume (TV; mm^3^) were calculated by assigning a threshold for the total bone content through CTAn analysis software (Skyscan). Then, the bone volume fraction (BV/TV) was used as a parameter to evaluate the bone formation ability (*n* = 6).

### Histological analysis

Following micro-CT scanning, the harvested craniums were immersed in 10% EDTA solutions for decalcification until the bone tissue could be readily pierced by needles. After being dehydrated in ascending concentrations of alcohols, the samples were embedded in paraffin wax. Four-micron-thick slices were generated by using a microtome. H&E staining and Masson trichrome staining were performed on selected slices from each specimen to assess bone regeneration. Images of the stained sections were observed by a bright-field microscope (Leica, DMi8, Germany). The percentage of new bone area of each specimen formed in the defects was measured via Image-Pro Plus 6.0 software (*n* = 6).

### Statistical analysis

Statistical analyses were performed using SPSS 23.0 software (IBM, USA). The data were presented as the mean ± standard deviation (SD) and were analyzed using one-way ANOVA followed by Tukey’s post hoc test. A *p* value < 0.05 was used as a criterion for statistical significance.

## Supplementary Information

**Additional file 1. S1** Synthesis of Ca_(10-x)_Co_x_Li(PO_4_)_7_ powders (x=0, 0.1, 0.25, 0.50, 1).** S2** 3D printing processes of Ca_(10-x)_Co_x_Li(PO_4_)_7_ scaffolds (x=0, 0.1, 0.25, 0.50, 1).

## Data Availability

The datasets used and/or analyzed during the current study are available from the corresponding author on reasonable request.

## Abbreviations

β-TCP: Beta-tricalcium phosphate
Co: Cobalt
rBMSCs: Rat bone marrow mesenchymal stem cells
HUVECs: Human umbilical endothelial cells
PDH: Proline hydroxylase: HIF-1α: hypoxia-inducible factor1-alpha
VEGF: Vascular endothelial growth factor: SE-3DP: slurry extrusion-three dimensions printing
ALP: Alkaline phosphatase
BMP-2: Bone morphogenetic protein-2
OCN: Osteocalcin
RUNX2: Runt-related transcription factor 2
