# Supplementary material for: Cobalt-doped bioceramic scaffolds fabricated by 3D printing show enhanced osteogenic and angiogenic properties for bone repair
Source: Biomed Eng Online. 2021 Jul 24;20:70. doi: 10.1186/s12938-021-00907-2 (PMC8306242; doi:10.1186/s12938-021-00907-2)
Supplement: Supplementary file 1 — Additional file 1. S1 Synthesis of Ca(10-x)CoxLi(PO4)7 powders (x=0, 0.1, 0.25, 0.50, 1). S2 3D printing processes of Ca(10-x)CoxLi(PO4)7 scaffolds (x=0, 0.1, 0.25, 0.50, 1). [file 12938_2021_907_MOESM1_ESM.docx]

**S1**

**Synthesis of** **Ca_(10-x)_Co_x_Li(PO_4_)_7_ powders** **(x=0, 0.1, 0.25, 0.50, 1)**

Crystalline Ca_10_Li(PO_4_)_7_, in which Ca was substituted with different contents of Co, was synthesized by the solid phase sintering method with Ca(NO_3_)_2_•4H_2_O, (NH_4_)_2_HPO_4_, Li_3_PO_4_ and CoCl_2_ as precursors. The corresponding chemical formulation was Ca_(10-x)_Co_x_Li(PO_4_)_7_, in which x= 0, 0.1, 0.25, 0.5 and 1 mol%.

Specifically, in a 30 ℃ water bath and under the magnetic stirring, the solution A containing 86.19 g (NH_4_)_2_HPO_4_ and 200 ml deionized water was dropwise added into solution B involving 220.19 g Ca(NO_3_)_2_.4H_2_O and 300 ml deionized water at the speed of one drop per second. After stirred 3 h continually, the pH value of the mixed solution was maintained at 7.5 ± 0.1 with NH_3_•H_2_O by a pH meter (PB-10, Sartorius). White powders were finally sintered at 800 ℃ for 1 h. The resulting white powders were β-TCP. 10 g CoCl_2_•6H_2_O was dissolving in 50 ml deionized water, then it was added into saturated (NH_4_)_2_HPO_4_ solution. The mixed solution was stood for 24 h, and then centrifuged at 5000 rpm and dried. The red powders were obtained as CO_3_ (PO_4_)_2_.

Following, the β-TCP, Co_3_(PO4)_2_ and Li_3_PO_4_ powders calculated by Ca_(10-x)_Co_x_Li(PO_4_)_7_ (x=0, 0.1, 0.25, 0.50, 1) were immersed in ethyl alcohol and grinded for 4 h in a planetary ball mill under the frequency of 15 Hz (QM-3SP2, Nanda). The uniform purple powders were acquired by drying the ethyl alcohol slurry and passing through a 200 meshes sieve, and named Co1-CLP, Co2.5-CLP, Co5-CLP and Co10-CLP respectively. CLP with no substitution of Co for Ca, named Co0-CLP, was the control group and had the similar synthetic process.

**S2**

**3D printing processes of Ca_(10-x)_Co_x_Li(PO_4_)_7_ scaffolds (x=0, 0.1, 0.25, 0.50, 1)**

The slurry extrusion 3D printing (SE-3DP) techniques was used to fabricate porous scaffolds. 5 wt% PVA solution was procured by adding 5 g PVA into 95 g deionized water under 90℃ water bath and magnetic stirring for 2 h. Then, 25 g PVA solution was added into 30 g powders and under mechanical stirring, the mixture acquired was the Co-CLP slurries. The 50 ml syringe containing 20 G syringe needle with 0.61 mm inner diameter was used to control the size of slurry extruded from the syringe. The layer height of Co (0, 1, 2.5, 5, 10) scaffolds was sliced to 0.6 mm according to the inner diameter of the syringe needle. The slurry extrusion was controlled by the air pressure, which was performed under 0.3 to 0.5 MPa. To obtain optimal porous scaffolds, the slurry printing speed was set as 30 to 40 mm/s. Porous scaffolds, abbreviated as Co0, Co1, Co2.5, Co5 and Co10 scaffolds, were obtained and sintered at 950°C for 3 h.
